# New! F-18-based PET/CT for sodium-iodine-symporter-targeted imaging!

**DOI:** 10.1007/s00259-020-04763-5

**Published:** 2020-03-16

**Authors:** Frederik A. Verburg, Luca Giovanella, Martha Hoffmann, Ioannis Iakovou, Jasna Mihailovic, Petra Petranovic Ovcaricek, Alexis Vrachimis, Markus Luster

**Affiliations:** 1 Department of Nuclear Medicine, University Hospital Marburg, Baldinger Straße, 35043 Marburg, Germany; 2 Clinic for Nuclear Medicine and Competence Centre for Thyroid Diseases, Centre Imaging Institute of Southern Switzerland, Bellinzona, Switzerland; 3 Clinic for Nuclear Medicine, Zurich University Hospital, Zurich, Switzerland; 4 Department of Nuclear Medicine, Radiology Centre, Vienna, Austria; 5 Academic Department of Nuclear Medicine, Aristotle University, Thessaloniki, Greece; 6 Department of Radiology, Faculty of Medicine, University of Novi Sad, Novi Sad, Serbia; 7 Department of Nuclear Medicine, Oncology Institute of Vojvodina, Sremska Kamenica, Serbia; 8 Department of Oncology and Nuclear Medicine, University Hospital Center “Sestre milosrdnice”, Zagreb, Croatia; 9 Department of Nuclear Medicine, German Oncology Center, University Hospital of the European University, Limassol, Cyprus

Dear Sir,

Targeting of the sodium-iodine-symporter (NIS) with radionuclides stood at the birth of nuclear medicine [1]. Ever since, it has been a cornerstone of the work of nuclear medicine physicians throughout the world. The theragnostic principle was pioneered using the direct NIS substrate iodine: its various isotopes could be used for imaging (I-123, I-124 and to an extent I-131) and therapy (I-131) alike. In no way would the biological behaviour of the substances (or in this case: isotopes) used for imaging differ from the one used for radionuclide therapy, making it perfectly possible to select and direct radioiodine therapy based on radioiodine imaging. Somewhat later, a different NIS substrate was developed with more suitable properties for imaging in terms of a shorter half-life, lower radiation burden and a radiation spectrum more suited for use with gamma cameras: Tc-99m-pertechnetate. This made thyroid scintigraphy possible at low cost and comparably high resolution.

For a long time, this spectrum sufficed for all thyroidological imaging needs. Even for positron emission tomography (PET)-based imaging, iodine 124 could be used [2, 3]. However, just like I-131 poses problems for gamma camera imaging due to high energy radiation spectrum, I-124 does the same in PET. Furthermore, again like I-131, the long half-life of I-124 limits the activity that can be administered compared with other F-18-based PET tracers. To add insult to injury, I-124 is only available at high commercial prices or requires a powerful cyclotron to produce. Thus, nuclear thyroidology for a long time has been lacking the high sensitivity, high-resolution imaging possible which is available for so many other targets in physiology using F-18- or Ga-68-based tracers.

Enter [^18^F]tetrafluoroborate ([^18^F]TFB). As early as the 1950s, [4] reported the radiolabeling of [^18^F]TFB by isotopic exchange using reactor-generated [^18^F]fluoride on non-radioactive TFB under acidic conditions. For reasons unknown to us, this agent was not identified as a tracer with particularly high thyroidal uptake [5]. However, in 2010, Jauregui-Osoro [6] in a preclinical model of normal thyroid tissue and thyroid cancer showed clearly that TFB is a first-class NIS-targeted physiological imaging agent, showing excellent uptake both in normal rat thyroid cells and in a model of papillary thyroid cancer. The same group then in 2017 [7] published the first results of [^18^F]TFB evaluation in human subjects, showing a biodistribution similar to pertechnetate and a low effective dose of 0.0326 mSv/MBq in five patients with differentiated thyroid cancer (DTC), which is in line with other F-18-based tracers. These results were confirmed by Jiang et al. [8], who found an effective dose of 0.036 mSv/MBq in males and 0.064 mSv/MBq in females in a total of four healthy volunteers. Then, Samnick et al. published first results of [^18^F]TFB PET/CT acquired before initial post-surgical radioiodine therapy in a pilot study of 9 DTC patients [9], and compared the results with post-therapy whole-body scanning (dxWBS). Aside from some minor differences likely caused by the different timing of image acquisition after tracer application, this study showed that results were in excellent agreement.

In this issue of the *European Journal of Nuclear Medicine and Molecular Imaging*, Dittmann et al. [10] now have compared [^18^F]TFB PET/CT in the much greater number of 25 DTC patients with the results of whole-body scintigraphy with a diagnostic I-131 activity (dxWBS) as well as with 2-deoxy-2-[^18^F]fluoro-D-glucose (2-[^18^F]FDG). It has been well known for a long time that dxWBS has a much reduced sensitivity for DTC imaging compared with post-therapy whole-body scintigraphy with much higher therapeutic activities. This has many a time hampered the acceptance of results of imaging, with patients sometimes receiving a therapeutic activity for the sake of identification of thus far unknown foci of DTC. Confirming the low sensitivity of dxWBS, [^18^F]TFB showed a significantly higher rate of lesion detection: in 52% of patients, a recurrence or metastasis was found versus only in 12% on I-131 dxWBS. Furthermore, they found that, in agreement with previous literature on the so-called Flip-Flop phenomenon [11–14], the highest sensitivity (64%), positive predictive value (100%), and accuracy (64%) were obtained when combining the non-specific 2-[^18^F]FDG-PET/CT with specific, NIS-targeted [^18^F]TFB PET/CT.

Indeed, the latter study as well as previous ones implies that the sensitivity of [^18^F]TFB PET/CT is at least on par with dxWBS, but at a much lower cost in terms of undue radiation exposure of non-target organs. Certainly, for diagnostic purposes not only the much higher accuracy than dxWBS but also a lower radiation burden than dxWBS, the avoidance of any possibility of stunning of relevant lesions before I-131 therapy, the possibility of performing highly accurate diagnostic imaging in an outpatient setting, better comparability of images with concurrent 2-[^18^F]FDG-PET/CT imaging, and the (compared with I-124) ubiquitous and comparatively low-cost availability of F-18 for tracer synthesis are all arguments speaking in favour of [^18^F]TFB.

In an elegant stroke of “luck”, tetrafluoroborate furthermore is, in fact, a substance which is already used in medicine—even in nuclear medicine: it is a component of the formulation of kits for the production of [^99m^Tc]Tc-sestamibi, commonly used for myocardial and parathyroid imaging. In fact, for example, the EMA registration [15] of Sestamibi explicitly states: “…. Contains … [tetrakis (1 isocyanide-2-methoxy-2-methylpropyl-)copper(I)] *Tetrafluoroborate*”. Therefore, it can be assumed that TFB is safe and should not meet much in terms of resistance where it comes to potential registration as a medical product.

What still remains a hindrance for [^18^F]TFB is the undisputed fact that only very few individuals have been scanned with [^18^F]TFB—in literature, thus far, only four healthy volunteers and 39 DTC patients have been reported. While the results certainly look both promising and encouraging, the number of patients scanned still needs a considerable increase before any serious consideration and evaluation of the performance of [^18^F]TFB can be made in terms of generalized evidence-based recommendation, product registration, and indication for reimbursement by health insurance systems. Furthermore, studies have thus far largely focussed on thyroid cancer and less on other NIS-bearing cells. While Tc-99m-pertechnetate has been a reliable workhorse, it also has its limits, especially in anatomically more difficult localisations such as, e.g. retrosternal goitre or the localisation of a Meckel diverticulum. It is quite conceivable that [^18^F]TFB PET/CT will be able to provide an alternative imaging strategy here as well.

In spite of the difficulties and uncertainties detailed in the previous paragraph, the study by Dittmann et al. [10] is a beacon of hope for nuclear thyroidology. A hope that someday soon, nuclear thyroidologist will finally have a low radiation exposure, high resolution, high accuracy, PET/CT-based outpatient procedure for diagnostic imaging in DTC, and perhaps also in other thyroid and non-thyroid diseases. Certainly, the authors give this development the warmest of welcomes and can only encourage anyone interested to put in an endeavour to start studies further validating [^18^F]TFB, preferably in a multi-centre, international manner. Until such studies are complete, we can only wait with longing and yearning for that which for other imaging targets in nuclear medicine has been available for so long already.
